# Identification and characterization of the abscisic acid (ABA) receptor gene family and its expression in response to hormones in the rubber tree

**DOI:** 10.1038/srep45157

**Published:** 2017-03-23

**Authors:** Dong Guo, Ying Zhou, Hui-Liang Li, Jia-Hong Zhu, Ying Wang, Xiong-Ting Chen, Shi-Qing Peng

**Affiliations:** 1 Key Laboratory of Biology and Genetic Resources of Tropical Crops, Ministry of Agriculture, Institute of Tropical Bioscience and Biotechnology, Chinese Academy of Tropical Agricultural Sciences, No. 4 Xueyuan Road, Haikou 571101, China; 2 Life Science and Technology Center, China National Seed Group Co. Ltd., Wuhan 430206, China

## Abstract

Abscisic acid (ABA) is an essential phytohormone involved in diverse physiological processes. Although genome-wide analyses of the ABA receptor PYR/PYL/RCAR (PYL) protein/gene family have been performed in certain plant species, little is known about the ABA receptor protein/gene family in the rubber tree (*Hevea brasiliensis*). In this study, we identified 14 ABA receptor PYL proteins/genes (designated *HbPYL1* through *HbPYL14*) in the most recent rubber tree genome. A phylogenetic tree was constructed, which demonstrated that *HbPYLs* can be divided into three subfamilies that correlate well with the corresponding *Arabidopsis* subfamilies. Eight *HbPYLs* are highly expressed in laticifers. Five of the eight genes are simultaneously regulated by ABA, jasmonic acid (JA) and ethylene (ET). The identification and characterization of *HbPYLs* should enable us to further understand the role of ABA signal in the rubber tree.

Abscisic acid (ABA) is a classic plant hormone that was first identified in the early 1960s. ABA was originally proposed to be involved in abscission^1^ and has since been shown to play a role in plant growth and development, including cell division and elongation, embryo maturation, seed dormancy, germination, root growth, floral induction, and responses to both biotic and abiotic stresses, such as osmotic stress, chilling, high salinity, drought, pathogen attack and UV radiation^2^,^3^,^4^,^5^,^6^,^7^,^8^. In *Arabidopsis*, three core components within the ABA signalling pathway have been identified: the ABA receptor PYR/PYL/RCAR (PYL) protein family, the negative regulator type 2C protein phosphatase (PP2C) and the positive regulator class III SNF1-related protein kinase 2 (SnRK2)^9^,^10^,^11^. ABA receptors are a family of soluble proteins, referred to as PYLs, that constitute the beginning of the “core ABA signalling pathway”^3^,^9^. ABA binds to a PYL protein, resulting in inhibition of the phosphatase activity of PP2Cs and relieving the inhibition of SnRKs required to turn on downstream gene expression to convert ABA signals into the appropriate cellular responses^12^,^13^,^14^,^15^.

The rubber tree (*Hevea brasiliensis* Muell. Arg) is a tropical tree belonging to the *Euphorbiaceae* family. It is wildly cultivated to produce natural rubber (*cis* 1,4-polyisoprene). Natural rubber is obtained from the latex of the rubber tree. Latex is the cytoplasm of highly specialized cells known as laticifers in rubber trees^16^. Laticifers in the bark of a rubber tree are specific for rubber biosynthesis and defence against pathogen attacks^17^. Plant hormones play a crucial role in natural rubber biosynthesis. Laticifers are jasmonate acid (JA) responsive, as their differentiation is specifically induced by exogenous jasmonate^18^. Moreover, natural rubber biosynthesis in laticifers may be mainly regulated by JA signalling^19^,^20^,^21^. Ethylene (ET) is widely applied regularly on the trunk of rubber trees to stimulate latex production^22^,^23^. ABA treated rubber trees exhibited earlier significant response, leading to 4.3-fold significant increases in latex yield^24^, suggesting latex production maybe be also regulated by ABA signalling. SRPP is a major component of latex and clearly participates in the biosynthesis of natural rubber^17^,^25^. In *H. brasiliensis* the HbSRPP gene is regulated by ABA^26^, and in *Taraxacum brevicorniculatum* the TbSRPP gene was also regulated in an ABA-dependent manner^27^, indicating that natural latex production may be primarily regulated by ABA. However, knowledge of the ABA signalling pathway in the rubber tree is limited.

A draft *H. brasiliensis* genome sequence was recently reported^28^,^29^,^30^. The genome-wide ABA signalling pathway genes can now be identified and described. In the present study, a total of 14 PYL genes (designated *HbPYL1* through *HbPYL14*) were identified in the genome of the rubber tree. These genes could be grouped into three subfamilies that correlated well with the corresponding *Arabidopsis* subfamilies. High levels of *HbPYL* expression were found in latex. These genes were regulated by ABA, JA and ET.

## Results

### Genome-wide identification of the HbPYL gene family in the rubber tree

To extensively identify rubber tree *HbPYL* genes, we employed BLAST and hidden Markov model searches to search the rubber tree genome database using *PYL* sequences from *Arabidopsis* and rice as queries. After removing redundant sequences, a total of 14 HbPYL genes were identified in the genome of the rubber tree, and further conserved domain detection confirmed that all of the identified HbPYLs harbour a conserved hydrophobic domain and belong to the PYR/PYL/RCAR ABA receptor gene family (Table 1). We named these 14 HbPYL genes *HbPYL1* through *HbPYL14* according to their sequence similarities to *AtPYLs*. The identified rubber tree HbPYL genes encode proteins ranging from 187 (HbPYL11) to 224 (HbPYL7) amino acid residues. The other characteristics of the HbPYL genes, including gene length, open reading frame (ORF) length, the isoelectric point (pI), molecular weight (MW), and exons, are listed in Table 1. ORF length ranged from 564 bp (HbPYL11) to 675 bp (HbPYL7), MW from 20.77 (HbPYL2) to 24.35 (HbPYL7) kDa, and pI from 4.83 (HbPYL1) to 7.85 (HbPYL5). Most of these proteins (78.57%) have a low pI (pI < 7.0).

### Conserved residues of HbPYLs

**Figure 1:**
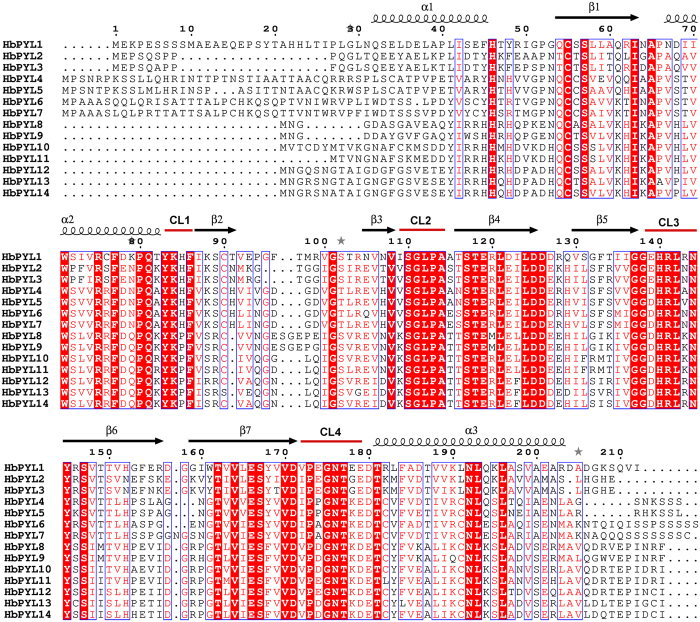
Sequence alignment of the fourteen characterized HbPYLs. Secondary structural elements are indicated above the primary sequence. Helices and strands are shown as black helices with squiggles and arrows, respectively. The four conserved loops CL1–CL4 are highlighted with red lines.

The results of amino acid sequence alignment indicated that the 14 HbPYLs shared a greater than 38.42% amino acid identity between pairs of sequences, with the maximum percentage of amino acid sequence identity being found between HbPYL10 and HbPYL11 (94.12%) (Table S1). All AtPYLs share a highly similar helix-grip structure characterized by a seven-stranded β-sheet flanked by three α-helices, and several conserved key amino acids have been found to be crucial for the function of ABA receptors. For example, the lysine located in the CL1 loop contributes to the interaction with ABA, and a highly conserved CL2 loop mediates the PYL-PP2C interaction. According to sequence alignment with reported AtPYLs, the HbPYLs all contain four identical conserved loops, which play important roles in ABA binding and PP2C interaction (Fig. 1).

### Phylogenetic analysis

**Figure 2:**
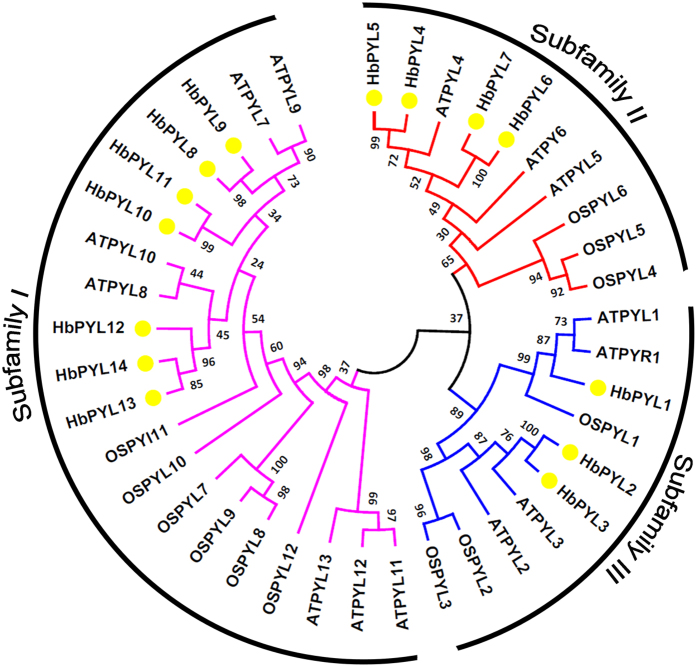
Phylogenetic analysis of HbPYL proteins from the rubber tree, *Arabidopsis*, and rice. A total of 14 PYLs from the rubber tree, 14 PYLs from *Arabidopsis* and 12 PYLs from rice were used to generate the NJ tree with 1000 bootstrap replicates. The HbPYL proteins are grouped into 3 subfamilies and distinguished by different colours.

To characterize the evolutionary history and phylogenetic relationships between HbPYLs and other known PYLs from *Arabidopsis* and rice, phylogenetic and molecular evolutionary analyses were conducted with MEGA version 6 by comparing 14 HbPYLs with 14 AtPYLs and 12 OsPYLs, and an unrooted neighbour-joining tree was created (Fig. 2). The results showed that the 14 HbPYLs could be assigned to 3 subfamilies, together with their orthologous PYLs from *Arabidopsis* and rice; HbPYL8–14, HbPYL4–7, and HbPYL1–3 were classified as subfamilies I, II and III, respectively.

### Gene structure and conserved motifs of HbPYLs

**Figure 3:**
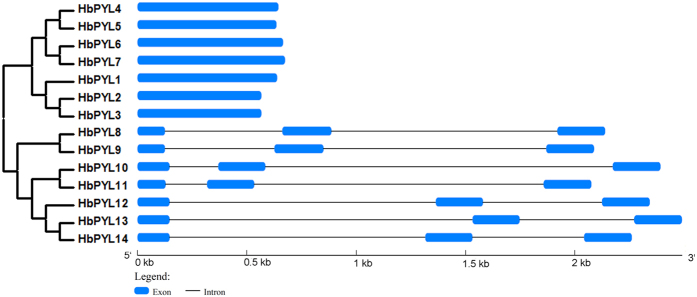
Exon-intron structure of *HbPYL* genes based on their evolutionary relationships. The NJ evolutionary tree was generated with 1000 bootstrap replicates based on the full-length sequences of the HbPYLs. Exon-intron analyses of the *HbPYL* genes were performed with GSDS. The lengths of the exons and introns for each *HbPYL* gene are shown proportionally.

To better understand the structural features of the HbPYL genes, intron/exon structure was detected based on their evolutionary relationships (Fig. 3). Gene structure analysis showed that the HbPYL gene members were clearly divided into a no intron clade and a two intron clade. No introns were detected in the HbPYL genes of subfamilies II and III, whereas all of the genes in subfamily I contained 2 introns, and these introns were typically flanked by GT and AG boundaries. In addition, the different HbPYL genes in each subfamily showed similar exon-intron structures, which supports their close evolutionary relationships and the classification of subfamilies.

**Figure 4:**
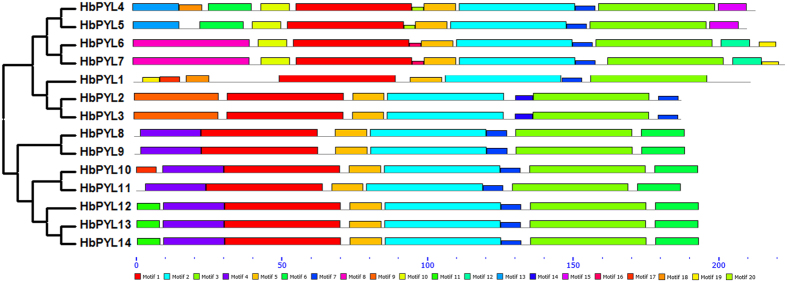
Conserved motifs of HbPYL proteins according to their evolutionary relationships. The conserved motifs of the HbPYL proteins were identified using MEME software. Grey lines represent non-conserved sequences, and each motif is indicated with a coloured box numbered at the bottom. The length of the motifs in each protein is shown proportionally.

Conserved motifs were also analysed for all 14 HbPYL proteins using MEME software. In total, twenty motifs were identified, and the details of each motif are shown in Fig. 4. The number of conserved motifs in each HbPYL varied between 7 and 11. Subfamily I contained more than ten motifs, whereas subfamilies II and III contained fewer than eight motifs. All of the proteins contained motifs 1, 2, 3, 5 and 7. Furthermore, all of the proteins in subfamily I contained motifs 4 and 6, while subfamily II harboured motif 10. These results indicate that HbPYL members clustered in the same subfamily show similar motif characteristics, suggesting functional similarities among members in the same subfamily.

### *Cis* elements of HbPYL promoters

To further elucidate the transcriptional regulation and potential functions of HbPYL genes, the upstream promoter sequences of the HbPYLs (750–1500 bp upstream of the initiation codon) were isolated and predicted (supplementary S1). A number of tissue-specific *cis* elements involved in the hormone stress response were found in the upstream sequences of the *HbPYLs* (Table 2). All of the *HbPYL* promoters except for that of *HbPYL1* contained two or more *cis* elements involved in the plant hormone response, such as an abscisic acid response element, auxin-responsive element, MeJA-responsive element, ethylene-responsive element, gibberellin responsive element, or salicylic acid response element. Additionally, all of the *HbPYL* promoters contained three or more *cis* elements involved in stress responses, such as low-temperature, heat stress, and anaerobic response elements. Cell- and tissue-type-specific expression elements, such as CAT-boxes, GCN4_motifs and Skn-1_motifs, were also found in the upstream sequences of these *HbPYL*s. These results suggest that there is diversity in the regulation of HbPYL gene expression in response to hormones and stress responses as well as for growth and development.

### Expression profile analysis of *HbPYLs*

**Figure 5:**
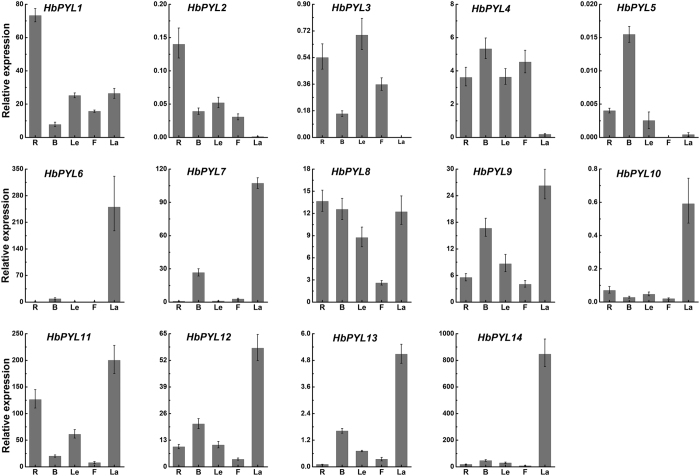
Expression patterns of *HbPYLs* in different tissues. The relative transcript abundance of *HbPYLs* was examined via qRT-PCR. The Y axis is the scale of the relative transcript abundance level. The X axis corresponds to the rubber tree tissues. R, root; B, bark; Le, leaf; F, flower; La, latex. Total RNA was isolated from roots, bark, leaves, flowers and latex. The rubber tree actin gene (GenBank: HQ260674.1) was used as an internal control.

To investigate the expression patterns of the *HbPYLs* in *Hevea*, qRT-PCR analysis of the *HbPYLs* was performed in rubber tree roots, bark, leaves, flowers, and latex. The tissue-specific expression profiles showed that all 14 *HbPYL*s were expressed in at least one tissue (Fig. 5). Five genes (*HbPYL1, 8, 9, 11* and *12*) were expressed in all of the tissues tested, although the transcript abundance of some genes in the tissues was very low. Four genes (*HbPYL2, 3, 5* and *10*) showed lower transcript abundance than the other genes in the above tissues. Eight genes (*HbPYL6, 7, 9–14*) were expressed at significantly higher levels in latex than in other tissues, whereas four genes (*HbPYL2, 3–5*) were expressed at very low levels or were not expressed in latex. In addition, pair-wise comparisons of the genes revealed similar expression patterns. For example, *HbPYL6* and *HbPYL7* showed much higher expression levels in latex, but *HbPYL2* and *HbPYL3* as well as *HbPYL4* and *HbPYL5* showed very low expression levels in latex. The subfamily I genes exhibited higher expression levels in latex, except for *HbPYL10*.

### The expression patterns of *HbPYLs* in latex respond to JA, ET and ABA treatment

**Figure 6:**
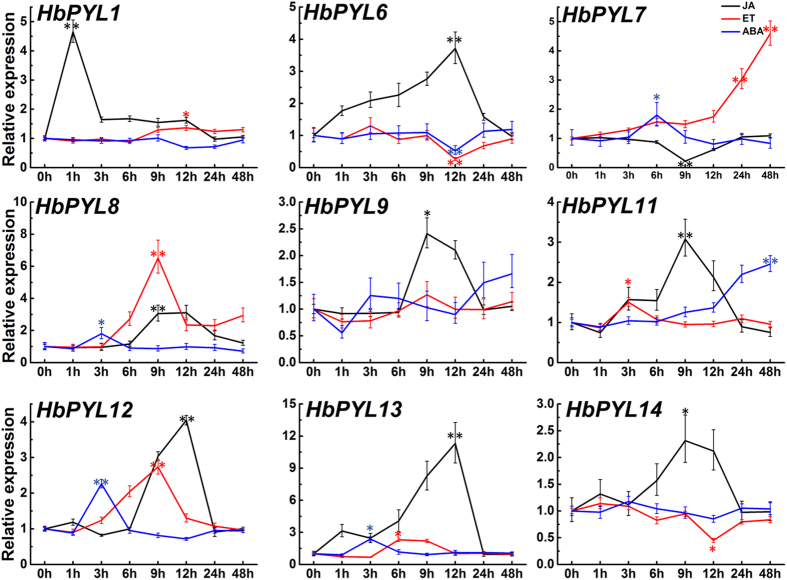
Expression patterns of the 9 *HbPYLs* responding to JA, ET and ABA treatment. The relative transcript abundances of *HbPYLs* were examined via qRT-PCR. The Y axis is the scale of the relative transcript abundance level. The X axis shows the time course of JA, ET and ABA treatment. The rubber tree actin gene (GenBank: HQ260674.1) was used as an internal control. Significant differences were assessed via ANOVA (one or two stars correspond to P < 0.05 and P < 0.01, respectively).

Ethrel is regularly applied to the trunks of rubber trees to stimulate the latex yield. JA is also a key factor related to the production of rubber trees. ABA is a ubiquitous hormone that regulates plant growth, development and responses to environmental stresses. The PYL proteins were identified as ABA receptors and are upstream of the ABA signalling network. To examine the expression patterns of *HbPYLs*, rubber tree shoots were treated with JA, ET or ABA. Nine genes (*HbPYL1, 6–9, 11–14*) were found to present relatively high transcript abundance in latex and were investigated after treatment with JA, ET and ABA. The results as shown in Fig. 6. All nine genes responded to JA treatment, 8 of which (*HbPYL1, 6, 8–9, 11–14*) were significantly up-regulated by at least 2.3-fold, while one (*HbPYL7*) was down-regulated 0.2-fold. Eight genes responded to ET treatment, 6 of which (*HbPYL1, 7–8, 11–13*) were significantly up-regulated by at least 1.36-fold, whereas two genes (*HbPYL6* and *HbPYL14*) were down-regulated (0.27- and 0.45-fold, respectively). Six genes responded to ABA treatment, 5 of which (*HbPYL7, 8, 11–13*) were significantly up-regulated by at least 1.80-fold, while one (*HbPYL6*) was down-regulated 0.52-fold. Among the 9 genes, 6 (*HbPYL6, 7–8, 11–13*) responded to all three treatments; 2 genes (*HbPYL1* and *HbPYL14*) responded to two treatments; and *HbPYL9* responded only to JA. Four genes (*HbPYL8, HbPYL11*–*13*) were up-regulated by the three treatments. *HbPYL6* was up-regulated by JA, but down-regulated by ET and ABA. By contrast, *HbPYL7* was up-regulated by ET and ABA, but down-regulated by JA. *HbPYL1* was up-regulated by JA and ET, and *HbPYL 14* was up-regulated by JA but down-regulated by ET.

### The genes involved in rubber biosynthesis in latex respond to ABA treatment

**Figure 7:**
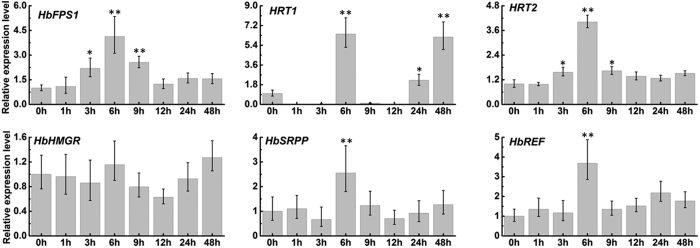
Expression patterns of genes involved in rubber biosynthesis in latex respond to ABA treatment. The relative transcript abundances of genes involved in rubber biosynthesis were examined via qRT-PCR. The Y axis is the scale of the relative transcript abundance level. The X axis shows the time course of ABA treatment. The rubber tree actin gene (GenBank: HQ260674.1) was used as an internal control. Significant differences were assessed via ANOVA (one or two stars correspond to P < 0.05 and P < 0.01, respectively).

Isopentenyl pyrophosphate (IPP) is the direct precursor for rubber biosynthesis and mainly derived from the mevalonic acid (MVA) pathway^17^. HMG-CoA reductase (HMGR) is a rate-controlling enzyme that catalyzes the reduction of HMG-CoA to MVA^31^. Post-IPP processes include initiation and elongation of rubber macromolecules. Farnesyl diphosphate synthase (FPS) catalyzes the biosynthesis of farnesyl diphosphate. The farnesyl diphosphate acts as the prime which is essential for initiating prenyl chain whereas Hevea rubber transferase (HRT), rubber elongation factor (REF) and small rubber particle protein (SRPP) are crucial for integrating IPP units into prenyl chain^17^,^31^. To examine the expression patterns of the genes involved in rubber biosynthesis in response to ABA, the rubber tree shoots were treated by ABA, respectively. As shown in Fig. 7, ABA markedly up-regulated *HbFPS1, HbHRT1, HbHRT2, HbSRPP* and *HbREF* expression, except *HbHMGR* expression.

## Discussion

In plants, ABA is a major mediator of plant stress responses and many developmental programmes^32^. Recently, PYLs has been identified as bona fide ABA receptors. PYLs together with PP2Cs, Snf1 and SnRK2s and downstream substrates constitute the core ABA signaling network. PYLs have been characterized in *Arabidopsis*, rice and tomato. For example, the PYL family consists of 14 members in *Arabidopsis*^13^,^15^, 12 members in rice^33^ and 14 members in tomato^34^. Most studies on ABA receptors have focused on those of *Arabidopsis*, whereas only a few of these receptors have been characterized in other plants, such as the rubber tree. The current report presents 14 members of the PYL family identified in the rubber tree based on publicly available data. These fourteen HbPYLs exhibited very high sequence similarities. According to the sequence alignment with reported AtPYLs. They all contain four identical conserved loops which play important roles in ABA binding and PP2Cs interaction^13^. The expression pattern of *HbPLYs* in rubber tree roots, bark, leaves, flowers, and latex indicates that some members clearly show a higher expression level compared with others (Fig. 5). *HbPYL6, 7, 9–14* showed the highest transcription level in latex, whereas *HbPYL 2*–5 were not detected in latex (Fig. 5). It was interesting to detect a high expression of several *HbPLYs* in latex since ABA signalling maybe to participate in latex and rubber production^24^. Some genes involved in rubber biosynthesis in latex were up-regulated by ABA (Fig. 6), and suggesting latex and rubber production may be regulated by ABA signal.

Although latex and rubber production appears to be polyphyletic in origin, similar mechanisms and underlying enzymes have been identified in all rubber-producing plants investigated thus far^33^. However, neither the detailed molecular mechanism of rubber biosynthesis nor the precise biological function of latex and natural rubber are completely understood. The transcriptome profiles of laticifers are relatively simple, with a high prevalence of rubber biosynthesis-related genes and high levels of defence-related genes, conferring the roles of laticifers in rubber biosynthesis and defence^17^. Latex and rubber production should involve several hormone signaling pathways such as ethylene, jasmonate, and ABA^35^,^36^. It was recently shown that JA and ET act as a signal molecule in rubber production^18,19,20,21,22^. Interactions of phytohormone signalings in regulating plant development and metabolism have been widely reported^37^. ABA and ET have antagonistic functions in the control of plant growth and development. In *Arabidopsis*, ABA activates the JA biosynthetic pathway. High levels of ABA are required to trigger the JA signalling pathway^38^. JA signalling involving the ABA receptor PYL4 regulates metabolic reprogramming in *Arabidopsis* and tobacco^39^. Interaction of the PYL6 ABA receptor with the MYC2 transcription factor links ABA and JA signalling^40^. In the present study, *HbPYL6*–*8,12,13* are simultaneously regulated by ABA, JA and ET, suggesting that these genes play a role in the interactions of the ABA, ET and JA signalling pathways. The identification and characterization of *HbPYLs* should enable us to further understand the role of ABA signal in the rubber tree. It will be of great interest to elucidate the mechanism by which latex and rubber production are regulated in laticifers by ABA, JA, and ET as signalling molecules.

## Materials and Methods

### Plant Materials and Treatments

Rubber trees (*H. brasiliensis* cultivar Reyan 7-33-97) budded on seedlings were grown at the experimental farm of the Chinese Academy of Tropical Agriculture Sciences, Hainan, China. The shoots treatments were attached to the rubber tree were treated with 0.5% ethrel (w/v), 0.1% methyl jasmonate (v/v) or 100 μM abscisic acid, according to Hao’s method^18^. Latex samples were collected at 1, 3, 6, 9, 12, 24 and 48 h after treatment, from 12 shoots at each interval and were stored at −80 °C until RNA extraction. For latex RNA extraction, latex was dropped directly into liquid nitrogen in an ice kettle. Rubber tree roots, flowers, leaves, and bark were washed with double-distilled H_2_O and then immediately frozen in liquid nitrogen.

### Genome-wide identification of the PYL gene family in the rubber tree

Multiple database searches were performed to identify PYLs in the rubber tree. The annotated (predicted) rubber tree genes and proteins were obtained from rubber tree genome data (DDBJ/EMBL/GenBank under the accession: GenBank: AJJZ01000000). The PYL family genes of *Arabidopsis* and rice were obtained from Phytozome V10.1 (https://phytozome.jgi.doe.gov/pz/portal.html#). The PYL cDNA sequences from *Arabidopsis* and rice were used as queries for searches against the rubber tree genome databases. Default Blast settings were employed, but low-complexity filtering and removal of redundant sequences were performed manually. All hits that were considered candidate sequences were analysed using the NCBI Conserved Domain Search database (http://www.ncbi.nlm.nih.gov/Structure/cdd/wrpsb.cgi) to confirm that each gene was a member of the PYL family. Based on the results of BlastP and BlastN searches in the rubber tree genome database, we obtained information on cDNA sequences and genomic sequences. The molecular weight (MW) and isoelectric point (pI) of each protein sequence were calculated using ExPASy (http://web.expasy.org/compute_pi/).

### Multiple sequence alignment and phylogenetic analysis

Multiple sequence alignments of the HbPYL proteins were performed using ClustalW2^41^. Further processing of the alignment files was conducted using ESPript 3.0 (http://espript.ibcp.fr/ESPript/cgi-bin/ESPript.cgi)^42^ with default parameter settings. An unrooted phylogenetic tree of PYL protein sequences was constructed with MEGA 6.0^43^ via the neighbour-joining (NJ) method, and bootstrap analysis was conducted using 1,000 replicates.

### Gene structure analysis and motif detection in HbPYL family genes

The exon-intron structuresof the HbPYL family genes were determined based on alignment of their coding sequences with thecorresponding genomic sequences, and a diagram was obtained using the Gene Structure Display Server (GSDS 2.0, http://gsds.cbi.pku.edu.cn/)^44^. Motif detection was performed with theMEME program (version 4.11.2, http://meme-suite.org/tools/meme)^45^.The parameters were as follows: number of repetitions, any; maximum number of motifs, 20; and optimum motif width, between 4 and 40 residues. For other options, the default values were employed.

### Promoter region analysis of HbPYL genes

To investigate the *cis*-elements in promoter sequences of *HbPYL* genes, genomic DNA sequences located at about 750–2300 bp upstream of the initiation codon (ATG) for all *PYL* genes were obtained from rubber tree genome data (DDBJ/EMBL/GenBank under the accession: GenBank: AJJZ01000000). The results were further confirmed by sequencing the PCR products with *H. brasiliensis* RY 7-33-97 genomic DNA as templates (Supporting file), and primer pairs used were listed in Table S2. Genomic DNA was extracted according to Dellaporta’s method^46^. The PlantCARE database (http://bioinformatics.psb.ugent.be/webtools/plantcare/html/) was used to analyze the *cis*-elements in the promoter regions.

### RNA extraction and gene expression assay via qRT-PCR

Total RNA was extracted according to Tang’s method^47^. First-strand cDNA was synthesized using the RevertAid™ First Strand cDNA Synthesis Kit (Fermentas, Lithuania). Real-time RT-PCR was conducted with the primers listed in Table S2. The rubber tree actin gene (GenBank HQ260674) was employed as an internal control. Real-time RT-PCR was performed using the fluorescent dye SYBR Green (Takara, China), and melting curve analysis of the amplification products was conducted using a Stratagene Mx3005P Real Time Thermal Cycler (Agilent, America). The real-time RT-PCR conditions were as follows: 5 m at 95 °C for denaturation, followed by 45 cycles of 8 s at 95 °C, 30 s at 58 °C, and 20 s at 72 °C for amplification. Three biological replicates were used per treatment or control.

### Statistical analysis

Relative RNA expression levels were determined using the 2^−ΔΔCT^ method^48^. The presented values are the means ± SE of three different experiments with three replicated measurements. An analysis of variance (ANOVA) was used to compare significant differences based on Fisher’s LSD test at significance levels of P < 0.05 and P < 0.01.

## Additional Information

**How to cite this article:** Guo, D. *et al*. Identification and characterization of the abscisic acid (ABA) receptor gene family and its expression in response to hormones in the rubber tree. *Sci. Rep.* **7**, 45157; doi: 10.1038/srep45157 (2017).

**Publisher's note:** Springer Nature remains neutral with regard to jurisdictional claims in published maps and institutional affiliations.

## Supplementary Material

Supplementary Information

## Figures and Tables

**Table 1:** Basic information of HbPYL gene family and their putative proteins.

Gene	Accession No.	Gene length (bp)	ORF length (bp)	Exon	Predicted Protein		
					Size (aa)	MW (kDa)	pI
HbPYL1	KX787820	639	639	1	212	23.43	4.83
HbPYL2	KX787821	567	567	1	188	20.77	5.94
HbPYL3	KX787822	567	567	1	188	21.03	5.94
HbPYL4	KX787823	645	645	1	214	23.08	7.82
HbPYL5	KX787824	636	636	1	211	22.88	7.85
HbPYL6	KX787825	666	666	1	221	24.07	6.71
HbPYL7	KX787826	675	675	1	224	24.35	6.92
HbPYL8	KX787827	2139	570	3	189	21.04	6.95
HbPYL9	KX787828	2088	570	3	189	21.08	6.41
HbPYL10	KX787829	2392	582	3	193	21.87	7.25
HbPYL11	KX787830	2076	564	3	187	21.30	6.86
HbPYL12	KX787831	2343	582	3	193	21.59	6.41
HbPYL13	KX787832	2490	582	3	193	21.20	5.28
HbPYL14	KX787833	2261	582	3	193	21.43	6.02

**Table 2:** The putative cis-elements in the promoters of *HbPYLs*.

Gene	Size (bp)	Number/Hormone Response Element	Number/Stress Response Element	Number/Other Element
HbPYL1	1523		1/ARE, 1/TC-rich repeats, 1/LTR, 2/HSE, 1/MBS	2/Skn-1_motif, 1/as-2-box
HbPYL2	2036	2/ABRE, 1/AuxRR-core, 1/P-box, 1/CGTCA-motif, 2/TCA-element	3/TC-rich repeats, 1/HSE	2/Skn-1_motif, 1/circadian
HbPYL3	2164	1/ABRE, 1/AuxRR-core, 1/GARE, 1/P-box, 1/ERE, 1/TCA-element,	1/ARE, 5/TC-rich repeats, 1/HSE, 2/MBS	1/AACA-motif, 1/Skn-1_motif, 2/circadian, 1/AC-I, 1/AC-II
HbPYL4	2020	1/P-box, 1/CGTCA-motif	2/TC-rich repeats 1/LTR 3/HSE 3/MBS	1/RY-element, 1/GCN4_motif, 3/Skn-1_motif, CAT-box
HbPYL5	2281	1/ABRE, 1/GARE, 1/CGTCA-motif, 2/TCA-element	2/ARE, 1/TC-rich repeats, 1/LTR, 4/HSE, 5/MBS, 1/Box-W1	1/RY-element, 1/CAT-box, 1/AC-II
HbPYL6	1180	1/TATC-box, 2/TCA-element	1/ARE, 1/TC-rich repeats, 1/HSE, 1/MBS	2/Skn-1_motif, 1/as-2-box, 1/CAT-box, 2/circadian
HbPYL7	2078	1/TATC-box, 1/ERE, 1/TCA-element	3/ARE, 3/TC-rich repeats, 1/LTR, 3/HSE, 1/MBS, 1/AT-rich element	3/Skn-1_motif, 1/as-2-box, 1/CAT-box, 1/O2-site, 5/circadian
HbPYL8	2163	1/AuxRR-core, 1/GARE, 1/P-box, 1/CGTCA-motif, 2/TCA-element	3/ARE, 1/TC-rich repeats 1/LTR 2/HSE 1/MBS, 1/Box-W3	1/GCN4_motif, 4/Skn-1_motif, 1/as-2-box, 1/CCGTCC-box, 2/O2-site, 1/AC-II, 1/A-box
HbPYL9	2275	1/AuxRR-core, 1/GARE, 1/ERE, 3/CGTCA-motif, 1/TCA-element	2/TC-rich repeats, 7/HSE, 1/AT-rich element	1/Skn-1_motif, 1/CAT-box, 3/circadian
HbPYL10	1760	1/AuxRR-core, 1/GARE, 3/P-box, 1/TCA-element	3/ARE, 1/TC-rich repeats, 1/HSE, 5/MBS, 2/Box-W1, 1/AT-rich element	2/GCN4_motif, 1/Skn-1_motif, 1/circadian
HbPYL11	769	1/TGA-element, 1/TATC-box, 1/P-box, 2/CGTCA-motif, 2/TCA-element	3/ARE, 1/TC-rich repeats, 1/HSE, 3/MBS, 1/Box-W1	1/GCN4_motif, 2/Skn-1_motif, 3/O2-site, 2/circadian
HbPYL12	1533	1/TGA-element, 1/GARE, 3/CGTCA-motif	2/ARE, 1/LTR 1/MBS	1/GCN4_motif, 1/Skn-1_motif, 1/O2-site
HbPYL13	2313	1/TGA-element, 4/GARE, 1/ERE	1/ARE, 2/TC-rich repeats, 1/LTR	1/GCN4_motif, 2/Skn-1_motif, 1/as-2-box, 1/O2-site, 2/AC-II
HbPYL14	2243	1/TGA-element, 3/GARE, 1/CGTCA-motif, 1/TCA-element	1/ARE, 2/TC-rich repeats, 3/LTR, 5/HSE, 1/MBS	2/GCN4_motif, 3/Skn-1_motif, 1/AC-I, 1/circadian

ABRE, abscisic acid-responsive element; AuxRR-core, auxin-responsive element; CGTCA-motif, MeJA responsiveness; ERE, ethylene-responsive element; GARE, gibberellin-responsive element; P-box, gibberellin-responsive element; TATC-box, gibberellin-responsive element; TCA-element, salicylic acid-responsive element; TGA-element, auxin-responsive element; ARE, element essential for the anaerobic induction; AT-rich element, AT-rich DNA binding protein; Box-W1/W3, fungal elicitor-responsive element; HSE, heat stress responsiveness; LTR, low-temperature responsiveness; MBS, MYB binding site involved in drought-inducibility; TC-rich repeats, defense and stress responsiveness; AACA-motif, endosperm-specific negative expression; AC-I/II, negative regulation on phloem expression; as-2-box, shoot-specific expression and light responsiveness; CAT-box, meristem expression; CCGTCC-box, cis-acting regulatory element related to meristem specific activation; Circadian, circadian control; GCN4_motif, endosperm expression; O2-site, zein metabolism regulation; RY-element, seed-specific regulation; Skn-1_motif, endosperm expression.
