# Supplementary material for: Identification and characterization of the abscisic acid (ABA) receptor gene family and its expression in response to hormones in the rubber tree
Source: Sci Rep. 2017 Mar 23;7:45157. doi: 10.1038/srep45157 (PMC5362889; doi:10.1038/srep45157)
Supplement: Supplementary Information [file srep45157-s1.pdf]

Supporting Information

Identification and characterization of the abscisic acid (ABA) receptor gene family and its expression in response to hormones in the rubber tree

Dong Guo[1]*, Ying Zhou[2]*, Huiliang Li[1], Jiahong Zhu[1], Ying Wang[1], Xiongting Chen[1] & Shiqing Peng[1]

[1]Key Laboratory of Biology and Genetic Resources of Tropical Crops, Ministry of Agriculture, Institute of Tropical Bioscience and Biotechnology, Chinese Academy of Tropical Agricultural Sciences, No.4 Xueyuan Road, Haikou 571101, China. [2]Life Science and Technology Center, China National Seed Group Co., Ltd., Wuhan 430206, China. *These authors contributed equally to this work. Correspondence and requests for materials should be addressed to S.P. (email: shqpeng@163.com)

Table S1. The percentage of HbPYL amino acid identity

	HbPYL2	HbPYL3	HbPYL4	HbPYL5	HbPYL6	HbPYL7	HbPYL8	HbPYL9	HbPYL10	HbPYL11	HbPYL12	HbPYL13	HbPYL14
HbPYL1	54.26	52.66	47.50	47.00	47.55	44.71	42.70	43.24	42.63	44.02	41.05	38.42	40.53
HbPYL2		92.55	50.00	49.44	47.83	48.94	43.02	43.02	40.66	42.22	42.86	40.66	42.31
HbPYL3			48.88	48.31	46.74	47.87	43.58	43.58	40.66	42.22	42.86	40.66	42.86
HbPYL4				87.68	67.98	69.61	47.75	48.31	48.09	49.72	46.45	43.72	46.45
HbPYL5					69.46	71.08	48.88	48.31	49.18	50.85	47.54	45.36	46.99
HbPYL6						88.64	46.45	46.45	46.03	46.99	43.92	42.33	44.97
HbPYL7							47.06	47.06	45.60	47.06	44.04	42.49	45.08
HbPYL8								91.53	79.57	78.49	77.96	72.58	76.34
HbPYL9									74.73	73.12	72.58	67.74	72.04
HbPYL10										94.12	75.65	72.54	77.72
HbPYL11											78.07	73.26	78.07
HbPYL12												87.05	90.67
HbPYL13													91.19

Table S2 Primers used for cloning of *HbPYL* genes,promoters and qRT-PCR analyses.

Usage	Gene	Forwardprimer(5'–3')	Reverseprimer(5'–3')
cloning	PYL1	CCCTATTTACCCTCGTCTTTTGT	GGCCCATCGAACAGCAGAAAA
	PYL2	TTCCTCAATGAAATGAACCCAA	CTATCACGGCTCCCGTCACCA
	PYL3	TCTTCTCCTCCATACTCTTGC	ACCCAGCTTCCATGCTAACCACT
	PYL4	ACAGCACCAGAAGTTATTAAACC	ACTCACATCGGATACTCACAAA
	PYL5	ACAGAGCCAAAAGTTAGTAAACC	ACACTCACATCCGATAGTAATAA
	PYL6	TAACCCTAAACGGCTAAACCA	CTAAGTTGGCAAAGCACAAAA
	PYL7	AACAATCCTCAAGAAACCAACCGAA	CTATAAGCACCTTCAAGAGCC
	PYL8	TGGCAGCGAATCCCTCTGTTT	TTCTGGGAATAGTGAAGGAGTA
	PYL9	CACCGAATCCCTCTTCTGTTGGACT	AAGGGTCTCCATGAGGTGAAT
	PYL10	TTTGGGTCACGATCTTCCTCT	ATAAACAGAACCCACCGCACT
	PYL11	AATCCAACTCCGCTCTTCTCG	GACAAGATACCCAAATGATGAA
	PYL12	TGTGATTTCGGCGAGGGAGGT	ACTACAGCCAGTCCGTATCCA
	PYL13	CGGCGATCCTACCAGTGATTT	CCTTTATACGGATGTGATTGTTGA
	PYL14	CGGCGAGGGAGAGTTTTGAGAGAG	GTACTAAAGCCAGTCAGTATCCA
qRT-PCR	PYL1	CGGTTTTACTATCATTGGCG	CGATTCTAAAACAACGGTCC
	PYL2	GATGTTTGTGGATACTGTTGT	TATCACGGCTCCCGTCACCA
	PYL3	GATGTTTGTAGACACTGTTATC	TCACCACCACCACTGGCAA
	PYL4	ATTACAGATCGGTGACAACT	AGGAATATCCACCACGTAAG
	PYL5	CCAGACGCCATAAATCATC	AAAACGACTGTGTATCACCA
	PYL6	AACTTGGAATCTTTGGCTCA	TCGAAGAACCCGTTTCAAAA
	PYL7	ATTGCTACCATTCTCGTACC	GCTTGAGGGTTATCAAAACG
	PYL8	CTTTGTGAAGGCACTCATCAAGT	CACATATTATGAAACCACCCATT
	PYL9	CTCTCATCCAGTGCAATCTC	GCACAGCCATCCTAATGAC
	PYL10	CTGGCTAATGTGTCGGAG	TAAACAGAACCCACCGCA
	PYL11	GTTCATCTTGTGTGGTCTCT	GGAAGTCCCGACTTAACATT
	PYL12	AGGATCGAACTGAACCCATTA	AGTCCGTATCCAGAGTGTC
	PYL13	CTCTGGTGATCGAGTCATTT	TCAGAGCTTCAACCAAGTAG
	PYL14	GTATTGTTGGTGGGGATCATA	CCACTACAAAAGATTCGATCAC
promoter	PYL1	TAAAGAATATAAACTAAAATTTTCTTAA	GACTCTGGCTTCTCCATAGAGAG
	PYL2	ATGCCTAAAATCATTAAGGCAATCGTTC	GGCTAGGTTCCATGAAGGACGATGA
	PYL3	CTTTTATCTGATTATCTGAGTATCGTC	CTTGGCTAGGTTCCATGAAGGAT
	PYL4	ATAAACTAAATACAATCTAATA	GATTAGAAGGCATTTCGTGCGG
	PYL5	AAATTGAGATATTTATGAAATATCAATAA	TTAGAAGGCATTTCTTGTGGGTGG
	PYL6	AAACTTTAATAATTTTTTAAAAATTTAAC	CTGCAGCAGGCATATTTGTGGGGTG
	PYL7	TTTAACATAATCTAAAATGATATTT	TGCAGCAGGCATATTAATTAGTGG
	PYL8	TCACACTATTACCATTTTGACTATTAT	CGCCGTTCATCTTTAACAAAATGA
	PYL9	ACTTTTATGAGGAGAGGTTAATTTTAG	ATCGCCGTTCATGTTTAATGATAT
	PYL10	CAATTAGGAAATCAATCAGATCTTGATAGC	GGTCACCATCCAGGCAACAAAAGAG
	PYL11	GCCAGCCTACAAGAGCAAGTTGC	TTGACGGTCATATAATCAGTGTTC
	PYL12	AAGTAATGACGTGCGATACATACTC	TGACCATTCATTTTCGGTTTGC
	PYL13	CTCAATTTTATTTCTACTCCGCCAT	CCATTCATTTTCGGTTAGCTTTC
	PYL14	ACTTGATATGAGAAGATAAAAAAGC	ACCATTCATTTTCGGTTAGCTTTCT

Supplementary S1 The promoter sequence of *HbPYLs* (The start code was red)

>HbPYL1

TAAAGAATATAAACTAAAATTTTCTTAAAAATATAAAAATAAACGGGCCGTAATTATTCAATCCTAGATTTCTTAAGAAATCATT
ATTGTTAAAAAAAAATCATCATTATTATAGGATTTTGCTAGTATGTACCATTTAATCGGAGTAGAAAACATGAGGTGACTGAAA
AAAAAAAAGAATAAGTAATACGTAAAGTGGAAGAGCATAAGAACCCATTAACAAGACAAGAAACGAAGGTTACCATTAGAC
AATATTCATAAATTATTAATTTTCTTTTAATGATTGTAACATAAAGAAAATCAACATTTGCAGTAGAAAAATTTACTATTTATTAA
TATGTTATCAAGGTTTGAGTCCATTTTTTAACTGGTGAAGGTATATAAATATATGTATATTATTTAATAAATATTATAGGTATAAAT
ATATTATTGAATTAGTTGTTCTTATAAAAAGTAATAATAATAATTAATTAATTAAATTATATCAAAGTTAGTAATTTAATTTGTTTT
ATAAGGAGATTACTTTTCACTTATAATTTTTCTTTTCTAAAACAATGTTATAAACTTATAAAATTAGAAAATATTGATATATTTCT
CATATTGTAAAAGGAATTAAGATAAAAAGAGTTAAATAATTAAACTAAATAATTATTGAAAATAAATTAATTAAGTGTGTATAC
ATATATATATATATAACTTATTATATTATGTATTATATGTATTAATTATTATTTTAATTAGAATATATATCTTTTAAAATCAATTTAATTA
AATTTAATATTTATTGTTTTAAAAAAATTTGAAAATTTTTAATTGTAATTTTATATTTTAAATGACAACATATTTTTATAATGTAGG
AAATAGCCGAAATAAAATTTTCCATAATAATAAATTTATTTAATGAAAAAAGTTACTTTTAAATTAAACTAATAACTAATTTGAA
CATTAAAATAGATTATTAATAATTAAAAAAATAATTAAGAAATAAAAGTTACTTTTTCAACCATTAAGCTAAAAAAAAAAGGAAT
CTAATCTAATTAACTTATCAAGGTAGCTGCACAATGCCCATCCCATGAAATCAGCGAGTGAAGCTAATCAAGAAGTTCAGAG
AAGATATGATGCAAACCAAAAAAAAAAAAGCAATAATAATAATTTTAATTTTTAATAATATTAAAAATGTTTTAAAAATTATAATA
TATTGAATTTAAATTTTAACTAAAATAAATTATATAAAATAAATGAAAATGAGTTCGGCTGTGTGGCTCAGTCAGATTGATCTC
GCAAGCAACCAACATGACTGAAACCCACCTGTCCACTTCACCCACTTGCCACCTTTCTTACATCTCATAAAACCTAATTTTTT
AATCCTTAAAATATTAAAAAAATCAAATATTGTAAATAATATAAATTAAAAAAGTTTAATTTCCCTATTTACCCTCGTCTTTTGT
ACAAAATCTCGCTCTTGTGTTGTATATCTAGAAAACTAAAGCCCCCGCTCCTCTCCTCTCTCTCTCTCTCTCTCTATGGAGA
AGCCAGAGTC

>HbPYL2

ATGCCTAAAATCATTAAGGCAATCGTTCACCAAAATATCTACAAGTGATTTTTTGCCTAGAATCTCCCATGGCAAAGGTTTACC
CAAAATCTCTAAGGTGATGGTTCACCCAAAATCTTAATGGCTCTATCACATGGCTAGAGCTTTGACGTCTAACACACAATTTG
ACACTAAATCTAAAGATTCATACTCGAGAGGGCATTAAGATACAATACACTCCAAAATACCCGAATGATCAGTTCAATCGATC
AAATGTCTAAATGATCAAGATTCAAACCATCTTAAGATTCAGATATACAAATTATTTAGACACCATGAGGTCTGCTTAATTGAA
TAAGAGTACTAGTCCAACATTAACTCTATTAAATAAGCATCCCACCTTATCTAAGAATTTTCTATTATCACACTCCTTGTAGTAA
CAGGACGCCCATTCAATACCTATAAAAGGGACCAATTTAAGACAATATTCTTTTTAGTTAATTATTTAATCTCAATTCCTATAAC
TAATTTAAGTATCGAAAATATTGCCAAACTAATCTATTGGAATTCTCTTTCTTGTTTCACAATTCCACACTCACAAGAGGTCAC
AGCAGCATCAAAACCTGAAGTGAGAAAACTTTAAAAGTCAATTTTGTTCTCTTTGAGCTTCAAATCAAAACTGTTAATTTAA
ATATGAAATTAGATTAAAATTGACGCACAGACAGGCCTAAGAAAAAGAATAGAAAGAAAATTAAAAAACAAAAATATGTGA
GCGTGAGGGAGTTGAGGCGCGGGGGAAATGGAAATCCAGCAAAGTGGGGTTATAATCACGCGATAGTTTGTTGGATTTGTC
ACATGACCTACGAATGTCCATCTACCCTTTTTAACGATGGCGACAACTCCCATCCTTTACCCATCTTGTCTTTTTCCCCCTTTTA
CCATTCTACCCCCACCTCTTTCTTTCTTGTATTGGATGCTATCACTAACAGCCGTCTCATTGCCCAACACTCCCGGTGGGAATC
AACTTACATGAATTTTATGGAACGAGAATTATTAAATAAAATCTCAATTGTTTTATTTTATTTTAATTAAAACTTAAATTTAAGAT
TTTATATTCATTATATCAGGATTTCAATTCTTTCTTTGCATTTCTTAAAAAATATAAAAAATAATTAATTTAACACGTATTATGAGT
TAATGAAATCTATAAATGTTAAGATACAAAAAATTCTTATCTATACTCGTTTCATTATAAACCCAAAATATAAATACGTGAGATG
TATATGTTTAATAATAGATTCTACCATATATTTTATATTTTAAATTTATAATAAATCATATTTAAATAAGAAAAATTCTTAGTATACA
TATATTCTTAATTGATAATTTATATAATTAAGGACAAGTGGGTCTGTCAAAATACAAAATAAAAAAGGAAAAAAAAAAAGAGT
AGGGTTAGGCAGAATCATGCAAGGTCGGACATAGAATGGGTAATAATTAGGAATGCGAATGCATTCTCATACTATATGTTATAA
ATGTAGGGATTAAAAAACAAGAAATGTTTAATAAAGTAAAGAGAGAATCCATTAAAAAGCACCGCCTAATCAACTCCCTTAT
GGCTCTTGGTAATAGGTCCATCTTCTAGCATTGCCTTTTGTCATGTTCCCAAAAGATAAGCACAAAAATATCTAATATTCATAA
CTCCCTAACTATCAATTACGCTTAACACACACACTTTCCCAATTATAGTTATCTTATTCACTTATTTAATTCTCTCACATTACAAG

TTAAACACATTTTCAAGGTAAGGAACTCACATGTCTGTTAAAAAAATTAATATTACAAGGGCATACCTGTCAAAAAACTCAAT
ATTAAATGTAAATAATCCCTACCCCATGTTCACATTATTAATTTGCTCTTTACCTAGCTATCTTATCTTTTTTCCTCAATGAAATG
AACCCAATTTTCTTCATTTCTTTTCCTCTTCTTCTCCATCGCCTCCATATTCTTGCTTGTTGAATTTGCTCAAAGCATACCCTAA
GTGCTCATCGTCCTTCATGGAACCTAGCC

>HbPYL3

CTTTTATCTGATTATCTGAGTATCGTCTGATTCAAACTGCTCATTATCCATTTATTCAGTTATTCATTACTTGTCTTATGGTATTAT
TTTATTGGTTATAATATTATTAATTTCATACCTCCCTCATCGAGGAATATTTATAAGCACTTATATTTCTACACTTATCTAATTTAA
AATCATGATAGGAGGAGAGTGGTACAAAATAACTTAGAAGCATTACAAATTTCCAAGGATTTAACCCAAAATTATTTAGAATA
GAGAAAGAAAATCCATATAGCCAACCTCAAATTATTAGGATAAAAGTTTTAGTTGAATTGAGTTAAATTGAGTTTAAAATCAT
GATATGATTTACTTATAAATATGAGATTATAATTTATAATTGATCGAGTGTATAAATTAATCTATACATTCTAATATAAAAAAGATT
TTTCCTTTAATTTTAAAACCAAAATGATAATCAATTAAATTACTTTAGTTTAATCACTCTCTTTCCTTTTTTTTAATTATTTACATT
TGTAAGAAAAATTAATACTATTTAAAATATATTTAATATTTCTAATAATTTAAATTTTTTAATATTATTGAATTAAAATTTATGAGT
GTCGTAATTTAAAATAATTATTTTTTAATGAATATATTTAATGGTCCACAATCAATGATGGACAATAATTAGTGATGATTAAAATA
TGGCATAAAATTAATAAATATAGAAATAAATTATATGGAAAGAAGAGTGAAAAAAAATAAGAAAATTAAAAAAAAAAAAGTG
AAAGTGGTCGAGTTGGGGGAAAAGAGATCCAGCAAAGTGGAGATATAGATCACGCGGGAGTTTGTTAGAGAAGTCACATGA
TCCACGAATCTCCAGCTTCCCTTTTCAACGATGGTGACAACACCTTTCATTTAACCCCTCTTGTCTCTTTCCCGTTCTATCCCT
TTCTCTTCTTTCATTCCCCTCCCTCTCCCTCCCTTAATCCCTCCCTATGTGGCTATATTTTATATTCTAAGTCCACTATAAATTTAA
AGTATAAAATATATAATAAAATTTATTTATTAAATATTTGAATATTATATATTTATATTTTAAATTTATAATAAATTAATTATAAATAA
GAATTTTTCTTAAATTTTATTACTTATCACTAATAGTTATTGCTTTGTTAATATATGAATTTTGGGTATGTGGGTTTAATAATTTAT
TTTTTATTTTCTTATAATTAGTTTTAGTTTGAATAAGTGATTCAAATTTAAAATTCTATAATTTTAAAGATAATTTAATATTATTAA
ACTAAAAACTCACTCGTTAATAATTTTTTTATTTGACAATAAAAGTCAATAAACACTCTCATTAAATTAAAAATTTGAGTATTA
CTAACTATTTCTTTTGACAAGCGATGATTAAGTTAAAAGTTAGAGTCTTACTAATTGTCCTTTTTGACAATGGATCTATTAACTC
CATTGTAATCACTTAGTAAAATTAGTTGTCCGCCTAAACCTTTTGAAATATGAAGGGGAAAATAGGGTGGGACAGAGTAGCA
AGGGACAATAATTCAGATGAGTAACTGTTGGAGGAAATGGTAATACTTATAGTGCGTTTCATGTAGAGATTAAAAAAAAAAC
AAAGAAATGCTTGTTAAAGAATAGTTTAAAAAGCACAACCTCGGGTCCCTTTAGCTCCTTGGTAATAGTTCCATGTTCTAGCA
TTGTCCTTTGTCATGTTCCTAAAAGATAACTACTCTTCACTCTAATTCAAAAGAATCTAAAGACTCTTAACTAACGCTTAACGC
ATACACTTTCCCAATATAGTTAATCTTAATGGACCACTTCTTAATTCTCTTATATTACAAGTAAACGCAATTTCAAGGTAATAATT
ATTCAACACAATCATTATGTTCTTACAATAAAAAAAAATTATTAATTCTGACATGTTGGATTGCCAAGGACATATATGTCAAATA
CTCTCAATATTATATATAAATAATCTATACCCATATTCACATTATTTGTTCTTTAGCTACCTATCCCTATCTATCGTGCCCTCAATCA
GATGAACCCATTTTTCTTCATTGCTGTTCTTTTTCTTCTCCTCCATACTCTTGCATGTTAAATTTGCTTTGAGCACCAAATAGAG
AAACGATATATAGGTCCTAACCCTAACTCATCCTTCATGGAACCTAGCCAAG

>HbPYL4

ATAAACTAAATACAATCTAATATTGTACTTTATTTATCTATATTTTTTAATAATTTTAGTTGTAAATTTATTAAATTATCTTATAATT
TTTAATTATAAATGTACTATCATATTATATATATGTTAATATATATGTTAATTCTTAATTATATTTATATCTTATAATTAAATATTATATA
ATTAATAAATAATTAAAATATATATTATATGATTTATTTATACTATTATATTATATAATATATTTAATTTTAAATTATATATATATTTTATAA
TTAAAAAATATATATAATTTAAAAATAATTAAAAGATGTATTACATGCATGTATATTATGTACTAATAATTTAATTTAAAATTTACA
TAATTATTTAAGTATGACTTTTTAAGAAAATAATTATAAAAAATTATTTTAGAAATAATAAAAATTAAATTAAAATTATATTAAAA
TTTCAGTTTAATTTTAATTCTTAATTAAACTCTAAATCAAACGGAAATCGCATGCTTCTATTTTTCCTAAAAATAATAATAAAAA
GAAACAGTTCATGGGAGAGTGGTGGATCTAATAAGAAGACCCAAAGGGCATAAAATGAACCCGGACCACCGATCAGAAGAT
TCGTACCTGTGGATCATGTGCCATGGGCTCCAACTTGTTCTTCTTTTTTACAATAATAATAATACTGCAACTGCAAGTGTATGA
AGAATAACAAGAAGAAGGCAATAAAAGAAGATAAAAGAATCACGTTCAAGATGCGAAAAGTTAAGATGCGAAAAGTTTTA
ACGGAAAGGCAAAAGCAACACGGCGGGGCTAATTTCATTGGAAGAGAGACAATATTAGACGCATGCAGCTTATATTGGCTAC
TTGCTAATTTTATATTATTTATTTATTATTCCTTTAATTGATTTTCAGTCTTCAGTCATTACCTGGTTCAGTTTTCATTTTTAATGCT
TATCCGATCCTTGGTCTTACGCCTCTGCATCCACTTGATCCTCCATTTGCTAGACTTTTTTCCCCATTCTCGCCCTCTCCTCTTC

CCTGTTTTACACATATGCCACTGCCTTTTTCCTTTTGATTGACTTTAATGCCCCCATCATATAGCATTACTCGAATTGGTCTTTTG

AACATGTGAACAGAAAAATAAATGTTGATTTTTTTTTATTTGAGCATTGAAGTTCTCTCTCTATAAGTTAAATATCTAAATCTAA

ATTCTTATAAATCTAATTTATTATTGATGGTAATATTTTTTTTATAATTAATTTTAATTAATATTTAAATTTGAAACTTCACAATTTA

AAAAATAATTTCAATATCATTAAATTAAAATTTATTAACATCAGTTAAGATAATATTTTTATTCATTGATTTTTTTAAAATCCTCTT

TTAATTATAAAAATATGAAATTGAGATTTTGCTATGCTTTGAATTTTTGATATTTTATTTAATTGACTTGGGGCTAAATAATACTAT

AGTAATCGTTTGAATAGGGCAAAATAATAATAATAATAACAATAATAATAATAATAATAACGTGCGTTTGAATTTGTTTTTTCTA

GCCAACGAAAGGCAACAAGTCAACAACTGTATTCATTATCCAATACGTTCAATTTCCCACGAGATAAAAGTAATAATAAAAAT

CATGCGTCATTCAGAAGGGTGTAATAGTAACTTAGAAAATTCAAAAGAAGGCTACTTTGGTAATTTCGGGACAAACTAGGGC

CCCTTTTTATTTGGGTTACCTTGCATGAATTTTCATCAACCTTCTTCTGCTTTAGAAAATCCAAATCACCAGCCCACCCCTTCC

CTCTCTCTCTCTCTCTCTCCCTCTCTCTCTCTCTGCAAAAGACATCAGTCGCTGCAAGTGCAAACAACACCACATTCACCTCT

TCCAGGTCGAGTTGGAAAGTTGCTAACCCCAATCTCCAGAATATATATACATACGCTGACACAAATATACAGCACCAGAAGTT

ATTAAACCTCAACAAACACCTGAAACCCATCTCTCCCTGTACATCACCACCCGCACGAAATGCCTTCTAATC

>HbPYL5

AAATTGAGATATTTATGAAATATCAATAAATAAAATTTTACTAAAAAGTAAAATGTATTAATAGAAAGTAATAGTAAAAATAATA

TAAATATATTTATTCTAAATAAAAATATATTATTTAAAAAAAAATAAAATAAAAAATCTTATAATTTCTATTAAAAAAATTTAGAG

TTATTTGAATTGAATTAAGTAATTTAGTTTCTCTACGTAAAGGAAGTGAGGTACTACGATCGAATGAAATTAATTTTTATACTTT

GTAGGAATTTGAAGTTCCTAACTGAATTATTTTTAACTACATAAGGGCTTAATATATATTTATCTTTAAAATTTATTATTTAAATTA

TAAAAAGGTTATATTTTTATTAAAATGAAATATTATTTTTATTTGTCACTAGAATATTGCATAATTTCTGAAATTGTTTTTGTTTAT

ACTCCAAAAGAAATTAAAAAAAAAAGAAGAAGAAGAAGGATTCTTAAATTTGGGTCTGCAGCTGCACGCGTTTGAAGAAG

AAGACGAGACAGTAGATAGAGAATCACATCAAAAGAAGTGAAAAAGTTATAAAGAAATGGTAAAACAAACAAGGCTGACG

CTAAGTTGGCGGCACATTGAAGAGTAGTAAATGTTTTGCTTAATTAAATTAAATTAAATATTTAATTTAATTTAACTACTTTCTC

TTCTTTAATTAATTTTCCTGTCTTTTAACTGATTCTGTTTTGTTTTGAATGCTTATCTTATCCCTCGGCTTACGCCTCTACACCCA

CTTGATCCTCCATTTGTTGCTTCGTTCTCTTTCTCGCCCTCCTCCTTCCCTTTATTTACCCATTTGCCACTGCCTTCCGTTGGTT

GGGAAATCCCTACTGTGGGATGTAATTTTACTCTAATGCCCTTTACATTTGTCAAAGAAATTTCGGGTCAATCTTTGCATCAAT

TTTTTTTTTTATAAAAATTCTTTGCATAAATTTAATTAATTTGAGACTTATTTTGTTATCAACGTATAATGTTTAAGTTAATTTAAG

ATAAAGTAAAAATCTTCTTTTTCTTAAAAAATTAATCACCCCAAATGATTAATTCTTTTAGCAAGTATACTAATTTTCGTTCATT

CTATCCAAACCTTGAAGAATCTATAAATTCAATCTTTTTCTTTATTTTATTCCCTTTATTTATAATTTTTATTTTTTTTAATTATTAG

ATTCATACGAAAGTTTGAACTCAGTATCTTATTACTTAAAAAATTGACTCTAATATTAAATCAAAACTTATTAATATATAATTAAT

TTTTTAGCTTTAATAACCATAAAAGTAATTTTATTATTTAAACTTGATTTATCATAAACTCAATACGTGTAAAATATATGATAAGA

TCCAAGAGAGCCTCTTCACCCTAAGAATATAATGAATTAAAACTCTTATATCAAGAGAAATTCTATCTAAATTCAGTTCATCAT

GAATAGAAATATAGATATATGAGATTTACTTATTTAATATGTGAATTTAACTATATACTATATATTTTGTGTTCATGATGAATAAAT

TTAGGTAAAAAAAATTATTGTATCACATTTTATTGATATATTCACTGAAAGAAATTATAAAATATAATTATTATATATATATAATAATT

TTATTATAATTATTAATTATGATGAGTTCTAAGTGAATTTATTATAATTAACAATTAAAATCTATTTGTTATACACATAATAATTGA

ATGCATGAAATAATTTATCTATTAATTTATATTTAATTAATTTAGGTGTAAATCTACTATGCTTTGAAAAAGGAAAAAGAAATAG

ACTTTGTTTTTTCTTAGCCAACCAAAGACAATAATTTGATTCACTTTCCAAAATTCAATTTCTTGGATTTAATTCCCAATAGGAT

AAAGTGATAAACCATGCATGAAAACATCAAGGTGTGCATCATATAGAAGGGTGTAATAGTAATTAAATACATTCAAAAAAAG

AAGGCTACTTTGGTAATTTCGAAAAGACCAACTAGGACCTTATCTTGTTAAATTGCCTTGCATGAACTTTCATTACATTCAACC

TTCTTCAGATTTAGAAAATCCAACTCGCCCATCCACCCCTCCCCTCTCTCTCTCTCTCTCTCTCTCTCATCGCCCACTGACAAC

AACATTCACCTCTTTCTAGTCTATTTGGAAAGTGATCATCCCCCAGCTTTTCCCTTCTCTCTCTGCGATATATGCATACAGAGC

CAAAAGTTAGTAAACCTCTACTCAAACCTAAAACCAATCTCTCCCCATTATAGATCGCCACCCACAAGAAATGCCTTCTAA

>HbPYL6

AAACTTTAATAATTTTTTAAAAATTTAACTCATTAAACATGTAAAATTTATTTTATTTTTTTTTATAATTAGTCTATTTAAAATGTG

TATAAAATAGAAAGTTTCCATTTTTTTAACCATCAATATTTTGAGAGTCATAAAGAGAAAAGAATACTGCTTTACGTAGCCCAT

AGACTCGCTTACCCTCTTTCTAATACACAATTTTTCAGGGTGCTGCAAAACTGAATGTGCAAATTGACCGATAAATTAAGAGA

AGAGATTTGACCTTTAATAAAATCATCATTATAATTAACTAAAAATGGCCAAAGAAAAAGAATGTAAAGTGTAATCTTAAGTA

CATTGATTCCAATCAAACAAAAAGAAACAAAGCCATGGTTCATGCGTTAGACCAATTGAAATTTGATTATACTTTATACTATCC

TTTCTCATATTAATTAGTTCACTGTAACATAACAGAGAATTTCTTGAAGGAAGAATCATCAAAGCATTAATTAGTATTTTTTTCA

TAGTTTAACATAATAAATTATTAAAACCTTCAACTTTATGAAATTAAAACCTTCAATATATTAGAGTTAATAAAAATAAATTAAA

AAAAGAGAGAACTTATTTAAGGAAAGTTAATCAAGTAAGCAAGTGAATATTTTTGCCATTAATTCTCCATATATAAAAGAAAC

AAATAGATTGAAAAATACCCTTTGTGGGGCTACAACTACCAAGAGGAAGTTGATTCCTTATTATTCATACACAAAAATATATAC

ATCATACATGTGATTGAGAAGTTGGCAAACCCTTTGAAAATGACAAAAATAAATTAAGTGGCATTCTTGTAGTTTTGAATTGA

ACAATGGGATAATCTACCTAAACTAAGTCCAAGCAACTTGCAAGTTGATGATCCACTTGCTTTAGTCGGGTGTATGATGCATG

ATAGGATCACCACCCCAAAAATCTCTCTCTCTCTCTCTCTCTCTCTCTCTCTCTCTCATCTCTTAAATCTCCAAGAAAACAT

CAAACTAATCCAAGTGGACATAAAGGAAAATTGAAATTTCTTTGCTTTCCCTTCTCTTTCTCTCTATATGCATATATATCTTCCC

ATGTCGTACGCATAATTCTTAACCCTAAACGGCTAAACCAAAACTGAAGAGACCAACCAAAAAGACAACAACAAACACCCC

ACAAATATGCCTGCTGCAG

>HbPYL7

TTTTTTATAAATTATTTTTTTAACATAATCTAAAATGATATTTTTTTCTTGAAAAGCAATTATACACTTTTAAATTTAATGTCAAAT

AGACACTAAAAAATACAAATAATAATTTTAATAATAACTTAACTTTTTATTTAAATTAATTTAGAAATTTTAATTTATGTTTAGTT

TAATATAAAAATTAAAATTTTTAGCTAAATTTCTTAATTTTTTAATTTAAATCAAAAATTAAGAATTTCTTGATTTTTTTCATTTT

ACCCAAAAATTAAGAAGATCGATTTCTTTTTTTTTTTTAATTTTTGAGGAAATTGAGTCTAAATTAAATTTTTTGAGTTAATTTA

AAAAGATTAAAAATAAATTAAATTTAATATTCCAATAAATATATGAAATTGAGCATTAAGTCTACAAAAATAGAGAGAAAAAA

AAAAGATTTTGATTAGATTATCAAAATAAATAAAATATTAATTAAAGTGTATAAAAAATAAGGATTTTTTATTTAAATATTCTTCT

ATATATTAATATGGTAAAAATTATAAAAAGAAGAAAAAGAAAAGATTGCTGCATGGTTTGTTGCCCATAGACTCAATTAACCT

CTGTCTTTTCTGGGGGCTGCATAAACTGAAAGTGCAAATTATAACCTATAAGTTTTGAATTCTTTTCTTTTCTTACTGTAAAAG

AGAAGAAATTTAACCCTTAATTAACAAAATCATCATTATTTTTACACAATGGCTGCCAAAGAAAAAGAATCTTATTTTAATTGC

TTGCCAATGGGGATGCTATTTATCAAGATTTCGATTTAAAGTGTAATCTTAAGTACATTGATTCCAATATATAAAAAATCCCATC

AAAATTAAAAGCCATGGTTCATGGATTAGACCATTAAAATGTGATTACACTTTATAATAGCTTGTCATGAAATAGTTTCACTTT

TTGATAATGTGATTTTGTTGTATTGATTCACTCTAAAGTATATATAGATTTTTTTTGCTTAAATTTGATCTATTATGAATTTAAGAT

ACAAAATATATGATAAGACTTATCTTTTAAATACATAAATTTTATATATTTATATTTTAGATCTATAATGAATATATCTAGTAAGAAT

TTCCAGCATATGCATATACAATGCACTATTGAAGAAGAATTATTAATTGCCAATGCCCATAAATTTCAAAGTCTAATTAACATAG

AGTTTGTAAAGATTTTGTTGGGTTTATTTTTATTTTTGTAATTAATATGAATTACGAAATAATTTTAATGATTAATTTAGTTAACA

ACTAGATTTTGTGCCAAATTTTGTTAATTAGAGTTTAAATATTAAAGTTAAATCAATAAGTATGTATATTTCTCATTATAAATTTC

TTAATTTCGATGGCCGGCGTGAATGGTCTACCTTTAATCAAACAAAAATGATGAACTTAATATCATCTCATCATAATAAAAAAA

AATTATGAAATTAAAATACTTATGAAAAAAAAAAATAAAGAGTAAATTTGTTCCTTCAAATTACTTAAAAAACTTTCAATTTTGT

GATGTTAGAAAGAAGAAAATTAAACCTATATTAGAAAAAGGAAATTAAGTATGCAATGTACATAGGAAAAGGAAATTGAGAA

GCAAATAGATTGAAAAATACCCTTTGTTTTATTGGGTTACAACTGCCAATGAGATGTTGATTCCTTAATTTCCATAAGCAAAAA

TATATACATGTCATTGAGAAGTTGGCAAACCTTTGAAAATGACAAACGAAAAAGTGGCATTCTTGTGGTTTTTAACGGAATAA

TGGGATAATCTACCTAAACTAAGTCCAAACAACTTGCAACTTGATGATCCACTTGCTTTATGATGCATATGATAAGATCACCAC

CCCAAAATTCTCTTTCTGATCTCTTAAATCTCCAAGAAAACATCAATTTAATCCAAGTGGACATAAAGGAAAATTAAAACTTT

CTTTTCTTTTCCTTCTCTTTCTCTCTATATACACTTTTATACCTCGCCTTTTCTTATGCATGATTCTTTTTATAACCCTAAAACAAT

CCTCAAGAAACCAACCGAAAAGAACACCCCCCACTAATTAATATGCCTGCTGCA

>HbPYL8

TCACACTATTACCATTTTGACTATTATTAAAGAGATCATTAAAATAATTTCTCTATCTTTCTTTAATATCTTCATCTTTCACCAATA

TTTTTCTTTCTTTATCCTTAATGCACCTAACTTGATTGAGATATTGACATTTCCTTTCTCTCCTCCTTACTAGTCTATAAATATCTT

TCTCCTTTTCTTTAGTTTTAAGTTTCTCATATAATTTTTCAAAGGCCTATGCTCTTGCTTAACTAACTATCTTTTTTGCCTATTTAT

TTGCTGTCTTGTACTGTTCATATGCCTCATTATTATCACATTTAGGTAATTTCTTATACCATTCCCTTTTTTCTCTTCATTGCCTTT

TGTACTTCCTCATTCCACCACCATCTCTCTTTTGAGGGTGGTCCATGTCCTTTAGACTCTTCAAGTAATTTTCTAGCTACTTCTC

TAATCTTTGATGCCATTTGTATCCACATGTCATTGGCCTCCATATCCAGCTTCCATGCTTCAAACTCGAGAAGCTCATTTTTGA
ACTTCACTTGCTTTACTCCTTTGAACTCTCACCACTTTGTTCAAGCTACACTATTTTTTCTAACCTTACTTGAATTGTTCATAAA
CTCGACATCTAATACCACTAACCGATGTTGACTTGTTAAAGCCTCTCCTGGAATGACCTTACAATCCTTGCATAGAGCTCTATT
TGTCTTCCTGGTTAAGATGAAGTCAATTTGGCTTCTATATTGCCCACTTTTGAAAGTTACTAAATGTGACTCTTTTTTTATAAA
GTAGGTATTTGCTAGTATTAAGTCGTATGCCATAGCAAAATCCAGGATGCTTTTTCCCTCCTCATTTTGACTGCCAAAACCAAA
ATCTCCATGAACATTCTTATAACCTTACCTATTACTTCCTACATGTCCATTCAAATCTCCACAAATGAAAACATTCTCTTCATTC
GGTATGCTTTGTATTAAATCATTCATATCTTTCCAAAACCTTTATTTACTCTCACTATTTAGTCATATTTGTAGGGCATAAGTACT
AACTACATTTATTGTTTCTCCTTCTAGTATTAGCTTCACTAGTATAATTCTATCTCCTACTTTTTTTCACAGCTATTATAGCGTCTT
TCAATGTCCTGTTTATAATTATGCCCACTCCGTTCTTGTTTCTCTCCTTTCCGGCAAACCACTGTTTGTACCATGAATTATTCAC
TTCCTTGCTTTTCTCTCATACCCATTTAGTCTCCTGAATGTAAGCAATATTCACCCTTCTCCTTTCTAAGGTATCCACAAGCTAC
ATTAATTTTCTTTTAAGTGATCCAACATTCCAAGTACCAACCCTGATCCTTCTCCTATCCTGTTCCTTCCTAATTGGTCTCCTTC
TATGATATCTTCTATTATTTTCTATGTCTATCTTGTATTTTGTTCCACTATCTGTCTTATGGACTAACTTATTTTCCTACACCCGTC
CATGATGTGGGAAACATTACACCCGGGCGCGGGCATAGCGTGTCGCTTTCGGTGAACGCCCTATACCCTTGCATATTTCTCAC
TACACCCGGCTTTGATGTAGCGCGTCGTAAGTAGAGGACATCTCAACATTTATATCATTTGAATCCATATCATAAGGTGTGACG
AAATTTTTATACTGGTTATCATCTACCATAATCCTCTTCCTTTATCCGGACTTGAAACCGACTAAGTGCAAACTATTTAGGTGA
AATTTATCAATTTACAACTAAATAGACATATTAATAACTTTATTTAAAGGATGAAAATGTATAAGGTAGCCGCTAAAATGATCGT
ATACAAACTCTCTGGCTTGATTATTGTCATCTCAAATGGAATTAAAATTTGTTTTAAATTATTTTAATTTATTTTAAACTTAATTA
TTATTATTATTATTTTAAATTTAAAAGAGTAAATTATATTTTTTTATGTTTTGACAAACCCCACAAACACATATATATATTTTTTATA
TATTGTCGGTGGATTCATTGAGTTTTGATAGTCTACTAAGCCAATCTAGAGCTAAGCCAAAGCCCAATTTGCATGGCAGCGAA
TCCCTCTGTTTAGATTCTGACCAGAGGCCAAAAGCTTATCTCCATAGCAAACCATCGAGATTTCTCATCCAGACACAAATAAG
GCGACTTCTATCGAGTGATTCATTTTGTTAAAGATGAACGGCG

>HbPYL9

ACTTTTATGAGGAGAGGTTAATTTTAGATTAAATCAACAAATTTTGATAAATTACATAAAAAAATAAAAATAAACAAAATGTG
AATAAAAAAAAATATTTATTTTTTTTCATGTAACATCTTATTTATTTTATGATATATTGCCATTAATAAAATCACATATTAATTATTG
ATGTAAGTTTATTAATGATTTAAATCTTAAATTTTACTTATATTAATAATTTGGTTCTCATATTTTACTTTTATTAACAAATTTTTTT
CTCAAAATTCACTAGTTATAGGACCAATTTGTTAATAAAGTAAAATGTGAAAATCAAATTGTTAGTAAAAAAAAAAAAATTTAA
TAATCAAATTATTACTAAATTTACATCTAAATTAATTTGTGAGTTTTATTAAATTTCAATCGTTTTGTTGTAATTTACCATTTGTTT
TACTAAGCAAAATTATTAATATCACCTTCTAGAAAATGTGGGCATTTTTATAAAAGTATCATATTGTTTGTCTTTTTTTAAAAAA
AAAAAAATTCAATTGCATCAAAAGAGTATCTAATGTATTAAATCACATGTGCATTTTAAAAATTTAAAAATTATTTCAAAAAAT
ATTCATATATTTATATTTTAAAACTATCAAAAATTAATTTAAGATCTATTAATTTAAAAAAAAAAATATTAGGAAAAGTATTATTATA
TTTATGCTTCAAATTTTTTTTATTATATAATATATGTGAAATTATGTTAATGGCAATAGTAGCATATATAGAATCTTGCAAGTTTTG
CAAATCAAATTTCCATTTCTATATATTTATGAGAAATTGTAGTCAAATAATACAGAATAACTTTAAATTTGTTTGAGAAATTTCT
TCAATTGTTATTTGATTATTTTGAGGCGTCTAAATTATTTATTTACTACTCCATTGAGGAGATTGTTGTCCTTGTCCCTATACATA
TGGACAATCACATATGGACAATTTACAGTCCATGCTCTTTTACTCCCTCTTCATGTACAAAATAGATAATTGCAATATAGAAAA
CAACAATCAATCAATTTTTAAAAAAAAAAAAAAAGCTATGTGGCGTTTGGAAAGAGTTAATCTTTGAAGAATAATCTATACATA
GAAACTACTTAAATAAGTCTCACATTCAGTCAATACCTTATATTAATATATCTTAACATATACCCTTTGATACTACACTATGCCAT
TAATTTATGATATCTTTTAATATCACGAATTATCAATTAAATAATAAAAATTTTGTTATTAATAAATCGATATCTAAAGAACTTTAT
ATTACAAATAGAAATGTGAGGACTATCTCCTTATAAAATTCAAAATCTCTGGTTCTATAAAACTCAAAATCTAAAAATAAAGGA
TGAAATTTATCCTATTTATTATCTAAAAATGTTAATCCAATAATATTTGCTCCGTTTTCATTTATTGCCAAATGCCAAGTCGGCGA
ACCACTAAACATTAATGGCATTATTCGGTACATACTGCATTTCCAGATTGATTTTAATAAAATTTATTTATTTCCAGATTGTTTGG
AGGTGATGTAACGGGACAGTTTAGCCTCCAAAGCCTTTAGAGATGAATCATTTTTGTACAACCCTTCCCTGCAATATGTTTGT
ACGTTCTATATTTATCTCTAAAAAACGAAAACTCTGCCTCGACCTGAAAGGGCAATTTCGTCAGTCCACCTTCATATTTATCCG
AATGGAATGTATAATCGTCCTAACATTACCCCCACTTCGTCATGCTTTGACTTTAAAGAAAACCCACTAGCTTAGTGGTGGTC
ACAATTTACATGCTAAATGGATACCTTCCATTTACCTACCACCTTTACCATTAAATAATAATAATAATAATAAAAAAAATCATCG

GTATAGAATGGACCGTCACCGCCAACACTGCAAAGCCTCTGTCACGTTACGCATTTTATTCATCTTAAAAAAAAAAAAATTACC
GTCGTCGCAATGTACAAATAAATAATAATATTAAAAGCTTCATCTGATTGGGGAAAAAGGGACCCATCCAGGCACCAGACTC
AGCGTTGGGCTTTAAAAACAATATAAAATGAGGGTTGTCGGTGGATTCATTGATTTTTGATAGTCTGCTAAGCCAAGCTAAAG
CCAAACCGTTTTGCAATTTTCACCGAATCCCTCTTCTGTTGGACTGTGACCCTGGCCAAAAGCTTATCCCCATTCCTTAGCAA
CAAGTAATCTGATTTCTCATTCACAAACTCACAAACCGCCACTTCTATCCAGAAATTTATATCATTAAACATGAACGGCGAT

>HbPYL10

CAATTAGGAAATCAATCAGATCTTGATAGCGTATAAAAAAAAAAGCAACCTTGATATTAGATTTTGCATGTGCTTTCTTCATGTA
TATTTAGCTCTCTAAATTTGATGGGTTGAAGCAATAACGAAAATTGAATTTATAAACAGTGACAAAAATCAAAGTTTGGAGAC
ACAACAATAGTATAAACAACAACAAGAATCAAATTAAATTCAAAACCAAAAATGGAGACACAACAGTAGTATAAATAGAATT
AGAAATCAGAAAATCATACCTTCTTGACTCAACAAGGGATAATCTGATACACTTTAATATCACAAATCAATTCTACCTTGAGA
AAAAATTAGAATTTTAATTGATAAGCTCAAGAAGGAGACACAATGATGGCACGAAAATTGAAAAGAAATCACAGCTTCAATC
GAAATAAGAGCAAGAAAGTAAAATAAGGAAGATAACAGATTGAAAAGAAATATGTATTTTGATTCAAATGTGAGCAAGAAG
ACGATATAGTGAAAGACTACGTAAATATTAGAGTAGGGATATTTTAGTCAAAAAATAAAAAAAATATATAGAGCAATTCTAAAC
AGCTGCCATTTTATAGTTGATAGAATCTGCAACTGATAGGTATTCCATCAGTTGCCTTTCAGTTATTAGCTAATTTTTTAGAGTT
TTACCAAACACTTTGGTTTAGCTATTTGGATACTTATCAGCTATCAGCTGTGTCCAACAGCTGAACCAAATGCCCTTTAAGGC
TTACAAGCATGTTTATGGGTTCAATCTTTTCTTGGTTCCAACCTGTATATTCCAGTTGTTCTCATCTTCCTATAAAAGAAATAAG
GCTTTCTTCAAGAAACTTGAGCAGCAATCATTTCATGAAAATATCTCAATAGCATGTCACAGTTGCATGTCATTTGATCTCTCC
AGCAATTCAATTCAAGGTCAAGTTCCAAGTTGATTTTAATGTCTTCATAGCATGGGATTATTAATTACTTTCCCTTGTGGTTTTC
CTTCATTAGATGGCTCAAGTTCTTAAATGTTTCATTGAATAACTTCATTGGAATTGTTCACTCTGGCAAGACCAACAAATTTGG
AAAATCTGCTTTTCTTCACGGAGGTAGTTTCAATTTCACTTCGTACGAAACCCCATCAAAAGCTCCAAGTATCTCGATGCAAA
TGCGAATTTGGCATCAAGGCTAGGGTTAAAAGCTGAGGTACGCTTTCAATTCCTCAAAGAATTTTGGGAAAAGCTTGAGGCA
CTTGAGGGATTTGCCTTTTGATTTGGGGATATATGTTCTACTTAGTAGGGCCCACATGATGGACCATTTAAAAACTGTGTGATT
GACTGCAAATTTTGTTTAGACACCCAATTAATTATTTGATAAAAATTGGGCTTTTTTAGAGCCTTTTGCCGTATCTAATTTACCC
ATCTCTTCCATTAAAAATCTCTATCAGAGTCAGGACCAGGGACCAACCCCTCGAAAGTACGGATAAACAATATTTAAAAAAA
ATTGCAAGTTGCCATTTATCACAAATTTTAAATTCATTTGTAAACGAAGAAAACGATTAGATGGGAAGCTCTGGACGTTTAGC
TATTGGTAGAGCTGTCCGTGGAGGCGGTGCATTTTGGATGCTTCTGTTGAGGCCTCCTCCTCTTCCTCCTCCGCTCTCCGCTC
TCCGATTCACTCGTTGAGTTCTATCTCCGCTTTTCCTGGCGATCTTTTGACCTTCTTTTTTTGGGTCACGATCTTCCTCTCTTTT
GTTGCCTGGATGGTGACC

>HbPYL11

GCCAGCCTACAAGAGCAAGTTGCTTATCCAAGAGATTTATGGGCTTAAATTGATAACTTAGCTTAGCCCATCAGTTTCCTATTT
CCATCGAGGAGGTGAACGTGAAGGAAGAATCACGGGGAAGGGAGAACTTGTTCATCAATGGAAGTTTTTTTCCAATTTGAA
GAGCACAAAATTAAAATTGACTGGCGGGTGTTAGATGGATGAAGTGAATATCTGAGGACCTCATGAAGAAATGTTTGGTTTC
TCTAGTACTGGTCCAAACCATTATGGTAAAGGCCGCTATTACAATTAGCTAAGCTTTTGGGCAATTCCCAAGTCAAATTTATGA
ATATCTGGGCCCTTCAAATTCATTACTTCCGGTTTTGGATGCTGAAATTGGCTTGCGGGATGGATAATTTGATGACGACGAAC
GACATCGTTTTACTTAACGCAGTTATCCCATTCTCCACTGCTCCTCATTGAAAATCTCTATCAGGACCGGACTAGAGAGAGAG
AGAGACCAAAACAAACAAGATTAAAAAAATGTGCAAGTTGCCATTTAATTTCATTTTTTAAATGAACAAAACCATGAGATGA
GAAGCTCTGGACCTTTCGTTATTGGAGGAACTGCCAGTGATGGCGGTGCATTTTGGACGCTTCTGTTGAGACCTCCTCCAAT
CTCCTCTTACCGATTCAATCGTTAAATCCAACTCCGCTCTTCTCGGTGATCTTTTGACCTTCCTTTACCGGTCACGATTTTCTTC
TTTTTACGGTGAACACTGATTATATGACCGTCAA

>HbPYL12

ATTTAATAATTTTATAAAAATATTTAAAAAATTAAGTAATGACGTGCGATACATACTCAATAAGTTATCATCATTCGATGTCAATT
TGGAGAGACTTAAGAGACATTAGGTGGAAGAGAGCGCGTGGTGGTGGCTTCCTGGACCGTGTGGGCTCCAGAAAACCGAT
CTGATAAAACTATGCGAGAGGTAATGAACGTTCGAGAAGTGTGTAAGAGCTTCTTGGACCAAATCGGATTATATATATACTTT
AAAATTGATTTTTTAAAATAAAAAATATATTTATTATAAAATTAAGTTAATATAAGAATAAAATTAATTTATATTAAAAAATATATA

AAATTAATTAAAATATTTATATAAAATACATAAATTTATTTACAATTTAATTTTAATAGTTGATTGGTTGATTTTTATATAAAATATA

AAACTGCATTAAAAAAAAAACAAACAAACAATGAGACCCAAGCCAAAGTCTTCAAACCACATGTTACGTTGGCGAAATTGA

CGAAACAATTTTAAGAGGCCCCATTCGCTCAAAAGACTTTTTTTTTTCTTTTTATTTTTTAAAAATAGAACAAAAATATATAAG

TTGAAGATAAAATTAAGGATGTGCATGAATTATTAAATTAAGCGGATGAAATTTAATTAGCTTAATGCAAATGTGCTTAATTTTA

TTATATTATTAAATAATAATTTTTAAAATTTTTAATTATTGACATAATTTTATTTAATTTAATAGCTAAACTAATTAAAATAATTAAA

TTTTTATTATAATTTATTATTTAATATATTAATATTATATTATTTTAATTATGCATTGATATTAATTAATTAAAATAAAGATTGAAAAT

AATATGTCTTTATCTATTTAAATTAAATATGTCTTTATCTATTTAAATTAAGTTAAATTAAATAATTTTTTTAATTATTTTTTGGACC

CTTTAGTTACAATTCGGAACGCAGTCCACATCATAGCCTTGGCTCAAACGCACCATAAAAATCTGATCACAGTGTCGAACTG

CAAAAATAAAATATTCAAGGCTACTTTCGCCGGGGACTTGAAGTTCCCATAAAACGACAAACATGATGGCACAGTCGTAATT

AACTTCAATTCCGAGGTTTAATGTCCCAATAAAAATTATTTAAAAAAAATGACGGAAATTATATATTTTTTCTTTATTGTATTTTTT

TATTTATTTATCTTTTGTTATTTCAGTTGATTAGATAATAGAAAGGCTTGAGGTAGCTCTTCTATGGCGGTGAGTCTTGTACGCT

TCCTCATCTCCTCTTGACAAAAGAAAGGCGAAGAAAAGACTTTTAACACGAATTCCTTATTTCCATATTAAAAGCGGTCCTTG

GTGGAGAATTATCGTCGGCCGGCCGTCGCACCTGTGATTTCGGCGAGGGAGGTGTTTTGGAGGGAGAAAGCGGACTTTGTC

TCTCTCACACGCTTTCTCTCTATCCATCTGATGGTTCTGTTTGCAGTGAGGGAAAGATAAGGACGCGTTTGGTTTGAAGAGA

AAGCAAACCGAAAATGAATGGTCA

>HbPYL13

GAAATATATTTTTTTATAATTCTTTTTTTTTTCTCAATTTTATTTCTACTCCGCCATTAGCCCATTATTAACAGCTAATTATTAAAT

GCATTAAGAGTATTAAAAAAATAATACGCCTTTAAATATTTAAATCTTTTTATTGTCTATGAAATATAAAACTCATATTTTTTTTG

CTAATGAAGCATGTACTTGAAGAGTATCCAATAATCCCTTATCACCAACACCCATTGTAGCACTATATACCAGCTCATTTTTTG

GTTGTCTCCTATTCTTACTCCATCTCCTTCATCACTTTCTGTTCCATGTTTATTTCAAGTTTCCTTGCTTCCTCTCCTCTTGCACC

ATTAACCCCTCCACCTACATCATCCCAACAATCCATAATTCTGTAATTTAAACCCTAATTATAGATCTCACAATCCTCTTTAATT

CTTCAAAATTTTTCTCTCAATTATGGCCTTAAATCGCTCGATGGTTGAAGATCTTTATAACACTGAAATTGATAAGAAAATTAT

CACAATTAAAGAAGTTGTCCTTGAAATATATATTACATTGCAGGCGTGGCGTAAAGTTTACTTGATTACAAAAACAACGTTGA

ATATCTTGGATGTGGCTTTAAGTTGCACATGTTAACAGTTTCACTTTCTTGCTGCTTTCATCCATTCTCTCCATTACTGTTCGCG

ATGCTAGGCCTCATTCCCTCTCTCTCTTTATGTTATGTTGTTGTTTTTCATCTTGATCCTTTCTTTTTTCCTCTTATGTTTTAGACC

CAGCAATAGTTCTTCCACAATATGATGACTTAATTTGAATTATGTAATTGAAATTATTGATTTGCATATTATGCAATTATTTACAA

ATGGGAATTTATTGATTTGTTGTTTTTGAAATTTTACTAGCTTAAAGATAAAATGGAAGGAAAGGATAGCAGTCCAGTAAATGT

ACAAGAGCTTCCCAAAATGCAAAGCCAATTAACATGTATGACATCCTTATCGCAACCACACCATAAAATCTCATGCACCTGTG

AAAATTTCAAGCACAACAACCACCACAAAACGACAAATTGATGGCACAATCGTAATTAGCTTCAAAATCCAAGGTTCATTTC

TTCATAATAAATTAAAGTATGGAAATTATATATTATTTATTGAGATTAATTATTTAAATAATGTGAATTTAAAAATATTTTATTAAA

ATAATATAAAATTTAAATTATTTACTAAAATAATATAGTATCATAGAAACTATCAATTATATATATATATATATATATATAGGATAATC

TTCAAGTTGTGTTTTTGCTTAGATTTTTACATTAAGAAGCAAAAATATTACAATTAAGGTATATTTTTTAACTTTAATGTAAAAT

ATTATTGTGATAATTAATATGATATATTTATTTAATATTTTAATAATAACAATTTTATTAATATGCATATCAAATTCTGTGATAAATTT

AGTGTTATATATTTAAATTATTGTTATTAGTTAAAAATTTAAAATAATATGTTTATATGATGCCAAATATTGTGATTTTACTTTTTAT

GCTATTAAGTCTATGATAATTGAAAAAAATAAAATCAACATGCTTTTGCGAATATGTTGATGTAGTCTATAAATTTCTATAATATA

TATGATAACTTTTTGTAATTTTAGCGATAAAATTATCAATTTTCAAAAAGTTTGTAAAATAAAAATTGGAAAGATACTAAACTG

ATATTTTTATTAATATATATTGTACATGTAAAGCTTTATTTGAAAATTTTAATGTTATGCATGCAAAGCTTTGATAATTTTTTTTA

AATAAAAAATTAATTTAATAAATTTAATATTTCATTTTAATTATTGGATATAAAAATTATAAATATAAAATATAATTAAAATTTATAT

GTTATATTTACTAAATTTAAATTTACATTAAAGATTCAGTATTCTGTTGCGGGAATAAATTATTTTTTTTAAACCATCAAACACA

GTGCAAAACAACACTAGATGATAGAGAGGCCAGAAGTTGTTCTTCTGTTTGCGCTGAGGGTCGTACGCTTCTTCTTCTTCTAT

TGACAAAGAAACAGAGAAGACACCTACGCGAATTCTTTGTCTTTCTTTAAAAGGGGTGCTCGGCGGAGAGTTATCATCGGC

CGGCGATCCTACCAGTGATTTTGGCGAGGGGGAGTTTTGAGAGAGAAAGCGAACTTTGACTCTATTTCTCTCTCTCTCTCAC

GCACAAACTCACTCTCTTTCTCTCTCCATCTGATGGTTCTGTTTGCAGCGAGAGAAAGATAAGGACGCGTTTGGCTTGAAGA

GAAAGCTAACCGAAAATGAATGG

>HbPYL14

ACTTGATATGAGAAGATAAAAAAGCCATTTTTCAATGGAGAAAAGAGGTCTTCAAACCATATGGTAGGTTAGCAAAATTTTC
ATATGTAGACATAGCAGTTGAAAGACACCTACTATCATCCAAGGACCTTTTTTCGTATAACTTATTATTTAATTTTATTATTATTA
ATATTTTAATTTTATATTTTAAATATATATTATAGAGTTTTTATGACTTGATTTCATTAATCATTTAATTATTTTGCTCATTTATTATT
ATTTCTATTGTTTATAAAAAAATAAAAATTACATTTTTTTTTCAATAAAACATGTATTTCAAGAGTATCCAATAATCTCTCGTTG
CACCATATGCTAGCAGCTTCACTCTGTTGCTGCATTCACCCTGTCTCTCCATTACTGTTCGCGATGCTAGGCCTCATTCCCCCC
TCCCTCTCTATGTTATGTTGTTGTTTTTCATCTTGATCATTTCTTTTTTTCTCTCATATTTTAGACCCAGCAATAGTTCTTCGACA
AATATGATGACTTAATTTGAATTATGTAATTAAAATTATTGATTTGCATATTATGCAATCATTTGCAAATGGAAATTTGTTGATTT
GTTGTTGTTGAAATTTGACTGGCTTGAAGATAAAATGAAAGGAAAGGAAAAGATAGCAGTCCAATAAATTTAGAAGGGCTTC
CAAAATGCGAAGCCAATTAATATGTAAAATATCCTTATCGCAATCACACCATAAAATCTCACGCACCTGCAAAATTTTCAAGG
ATAGCAAACCCCCACAAAACGACAAATAGATGGCACAAGCGTAATTAGATTCAAATCCGAGGTTATTTTGCTCGTTATATAGT
TAAAGTATGGAAATTATATATTATGCAAAAAGACTTTTTAAACATTTTAATAATTATTTTTATTGATCTACTTAAATCCTTAGTAA
TTACTCTAAATTATTAATTTAATTGATTCATTTAAATTTTGATAATTACTCTTATAGTATTCAAAATGTATGACTTACACTTGATGG
ATAAATGATTAAAAATATATAAATTGCTATTGTAATACCCTCACTTTAGATAGTTCGTACGTACTACTATTTCGGTGATTAGCATT
TGTTCAGACAGTCAAAATGTCTGTAATTATATTTAAACTAGAGTAAGAAATCATAAATAACTTAAATAAAGATAAGAAAAATT
AAAAAAATTTGAGAAATTTTTTTTATATACATGAATTGAGTATGAATGAAAATGAACATGAACTTGAAATATATAGATGATATTC
GAAACGTTGAAATGGTTATGATGAATATTGATAAAATTTTTATTATAAAATATTATTAAAAATTTCAAGTAAATGAATAATAAAA
TACGTCAATTGAAAATAAATAAAAAAAATTTTATTAGTTTCTCTTTTAAAAATATAATTTATTTTTAAAATATAATAAAAATAAATT
ATTAAAAAAATAAAATAAGATAAAATAGGATACTTCGATATCGAATATAACACACATCACTCGATTACACACCAAGTAGAGAG
TGTTATAGTTATTATCATTTAGTATGTCTAGTACTAATATAGAAGAGTATCTTCATGTGTTCCAAGAGCTAGTGAAATTGGACAT
TTTGAATTCACATTTAACGAAATATACAGTATAAAGTGATGATTTATAAAGGCTAATTTTTAATTCATGGAAATTTAAATGAGTT
AATTAAATTAATAAAGACTTCCTTGAGTCTGAAAATAATTGTTAAGACTTAAAAAAATAATTAAATTAATAAAAATTTATTTAAT
TCTAATAATAATTATTAAAACTTAAAAAATAATTAAATTAATAAAAATTTATTTAATACTAGAAATAATTATCAAGGCTTAAAAC
ACTCTTTGCCTCTGTATTATTTATTTTGCGGTTCCCACTAATAATAATTTTTGTTTTTTTAGGCGAATAGATGATAGAGAGGCTG
GAGATTGTTCTTCTATGGCGGTGAGGGTCGTACGCTTCCTCTTCTTCTATTGACAAAAGAGAAACAGAGAGAAGACACCTAC
GCGAATTCCTTATTTCTTTCTTTAAAAAGGATCCTTGGCGGAGAGTTATCATCGGCCGGCGATCCCAACAACGATTTCGGCGA
GGGAGAGTTTTGAGAGAGAAAGCGAACTCTCTCTCTCTCTCTCTCTCTCTCTCCATCTGATGGTTCTGTTTGCAGCGGGA
GAAAGATAAGGACGCGTTTGGCTTGAAGAGAAAGCTAACCGAAAATGAATGGT
